# Comparison of automatic image registration uncertainty for three IGRT systems using a male pelvis phantom

**DOI:** 10.1120/jacmp.v17i5.6332

**Published:** 2016-09-08

**Authors:** Jeffrey Barber, Jonathan R. Sykes, Lois Holloway, David I. Thwaites

**Affiliations:** ^1^ Department of Radiation Oncology Nepean Cancer Care Centre Sydney NSW; ^2^ Department of Radiation Oncology Blacktown Cancer & Haematology Centre Sydney NSW; ^3^ School of Physics, University of Sydney Sydney NSW; ^4^ Ingham Institute for Applied Medical Research Sydney NSW; ^5^ Department of Medical Physics Liverpool and Macarthur Cancer Therapy Centre Sydney NSW Australia

**Keywords:** IGRT, automatic image registration

## Abstract

A series of phantom images using the CIRS Virtual Human Male Pelvis was acquired across available dose ranges for three image‐guided radiotherapy (IGRT) imaging systems: Elekta XVI CBCT, Varian TrueBeam CBCT, and TomoTherapy MV CT. Each image was registered to a fan‐beam CT within the XVI software 100 times with random initial offsets. The residual registration error was analyzed to assess the role of imaging hardware and reconstruction in the uncertainty of the IGRT process. Residual translation errors were similar for all systems and <0.5 mm. Over the clinical dose range for prostate IGRT images (10–25 mGy), all imaging systems provided acceptable matches in >90% of registrations when incorporating residual rotational error using a dual quaternion derived distance metric. Outside normal dose settings, large uncertainties were observed at very low and very high dose levels. No trend between initial offset and residual registration error was observed. Patient images may incur higher uncertainties than this phantom study; however, these results encourage automatic matching for standard dose settings with review by treatment staff.

PACS number(s): 87.55.km, 87.55.ne, 87.56.Da

## I. INTRODUCTION

The high uptake of in‐room kV imaging systems with medical linear accelerators in recent years has placed tools for image guidance and automatic image registration at the treatment console for routine use. CT–CT automatic image registration for CT‐on‐rails for prostate IGRT gave results with a similar mean to a group of human users with smaller standard deviation.^(1)^ Automatic matching algorithms have also been shown to be accurate for matching 3D cone‐beam computed tomography (CBCT) scans to planning CT scans, on both the Elekta XVI system (Elekta AB, Stockholm, Sweden)^2^, ^3^ and the Varian OBI system (Varian Medical Systems, Palo Alto, CA).^4^, ^5^

The algorithms used in the systems investigated in the literature^2^, ^3^, ^4^, ^5^ were based on a number of standard image registration algorithms such as mutual information algorithm, Chamfer matching, and correlation ratio.^6^, ^7^ Cui et al.^(8)^ investigated automatic image registration of images from three treatment systems in three independent registration software packages. They found differences in the order of several millimeters for both head and neck and prostate clinical datasets, with the differences in information between the datasets (such as z‐slice resolution) not accounting for the registration differences. Therefore they highlighted the need for careful review and quality assurance of IGRT registration for clinical trials. Previous studies^3^, ^9^ have shown the accuracy of XVI automatic image registration to have a dependence with imaging dose. Image registration performance is dependent on the algorithm and the quality of the image and there are some significant differences in how commercial IGRT systems reconstruct 3D image sets — such as the handling of scatter and the preprocessing used for image reconstruction.

The role of image quality on image registration performance has not been investigated in a consistent manner across all IGRT systems. In this study images generated in three commercial systems (Elekta XVI, TrueBeam Advanced Imaging (Varian), and TomoTherapy MVCT (Accuray Inc., Sunnyvale, CA)), over a range of image parameters, are compared using one common phantom and are automatically registered in a single software system, using one single automatic image registration algorithm. Registration accuracy was investigated with varying dose and to assess whether image information affects automatic image registration for clinical settings. The underlying uncertainties of the phantom study can guide understanding of the fundamental limitations of IGRT in an ideal patient.

## II. MATERIALS AND METHODS

Using a common phantom, CIRS model 801‐P‐B Virtually Human Male Pelvis phantom (Computerized Imaging Reference Systems, Inc., Norfolk, VA), a series of CBCT images with variable exposure were collected on each linac imaging system (see Table 1). All images were imported to the XVI software v4.5 and multiple initial offsets and automatic image registrations then applied. The residual registration error from the ground truth was analyzed per image series, with ground truth being taken here as the systematic error (mean registration error).

**Table 1:** Parameters of imaging systems.

*Treatment System*	*# Image Sets*	*Voxel Dimensions (mm)*	*Exposure (mAs)*	*Dose (mGy)*	Reconstructions	
GE LightSpeed RT CT (reference image)	1	1.25×1.0×1.0	400	‐	Standard	‐
Elekta Synergy XVI			68	1.2	Standard	Sharp
			204	3.5	Standard	Sharp
			340	5.8	Standard	Sharp
	7	1.0×1.0×1.0	660	11.6	Standard	Sharp
			870	14.8	Standard	Sharp
			1360^a^	23.1^a^	Standarda^a^	Sharp^a^
			2176	37.0	Standard	Sharp
Varian TrueBeam			89	3.1	Standard	Smooth
			227	7.9	Standard	Smooth
			334	11.7	Standard	Smooth
	8	2.5×0.8×0.8	669	23.4	Standard	Smooth
			842	29.5	Standard	Smooth
			1070^a^	37.5^a^	Standarda^a^	Smooth^a^
			1338	48.2	Standard	Smooth
			2119	74.2	Standard	Smooth
Tomotherapy Hi.ART	3	6.0×0.8×0.8	Coarse	6.9	Standard	–
			Medium^a^	8.4^a^	Standard^a^	–
			Fine	19.6	Standard	–

^a^ Standard clinical presets taken as benchmark for comparisons between image series.

A single reference image set was acquired on a kV fan‐beam CT, GE LightSpeed RT scanner (GE Medical Systems, Waukesha, WI) with 1.25 mm slice thickness. For each imaging system, the phantom was set up and positioned using the IGRT system based on the reference image set. The initial phantom position was corrected in six degrees of freedom (DoF) on the Synergy using a Hexapod couch and 3 DoF on the TrueBeam and TomoTherapy using the standard couches.

Once positioned to within 0.5 mm and 0.5° of the reference scan, images were acquired with exposure (mAs) settings chosen to cover a range above and below that used typically in clinical practice. Where possible, the settings chosen were matched to be as similar as possible between systems (Table 1). Effective dose for each scan was calculated using published in‐air dose/mAs factors.^(10)^

### A. TrueBeam images

A series of eight CBCT images were collected on a Varian TrueBeam with Advanced Imaging v2.0 and exposure varied from 89 mAs to 2,119 mAs. Each image had 660 projections. After initially finding decreased image registration performance with TrueBeam images acquired with very high dose levels, TrueBeam images were reconstructed with both standard and smooth settings, denoted ${\text{TB}}_{\text{sharp}}$ and ${\text{TB}}_{\text{smooth}}$, respectively, to investigate the effect of image noise on registration performance.

### B. XVI images

A series of seven CBCT images were collected on an Elekta Synergy linac with XVI v4.5 with exposure varied from 68 to 2,176 mAs. Scatter in the XVI projection images was corrected by a background subtraction. Since the XVI images with the default reconstruction settings (${\text{XVI}}_{\text{smooth}}$) were inherently smoother than the Varian images, projection data were reconstructed and analyzed with both the standard reconstruction, denoted ${\text{XVI}}_{\text{smooth}}$ and a sharp reconstruction (median prefiltering disabled) denoted ${\text{XVI}}_{\text{sharp}}$.

### C. TomoTherapy Hi·Art images

A series of three MVCT images were collected on a TomoTherapy Hi·Art (Accuray) with one image acquired with each of the three pitch settings: coarse, medium, and fine. With the TomoTherapy Hi·Art system it was not possible to alter the exposure directly. Adjusting the pitch of MVCT scans changed the longitudinal resolution of the reconstructed images, and indirectly affected the total scan dose. As TomoTherapy uses MV fan‐beam, rather than kV cone‐beam, no additional image processing was investigated as it was considered to present significantly different information.

### D. Automatic image registration

All images were imported in to the XVI software for registration (TrueBeam and TomoTherapy DICOM images were converted to SCAN format files first). For each image set, an initial random six DoF misalignment of the CBCT/MVCT image was applied relative to the reference CT image, in the XVI program. Then the correlation ratio registration algorithm (“grey‐value match”) was run to register a masked region with six DoF. This mask encompassed the delineated phantom prostate volume with an additional 5 mm expansion and bone/gas excluded, per clinical protocol. The registration result was taken as the residual error of the registration algorithm. This process was repeated 100 times for each image using random initial offsets sampled from a 3D Gaussian distribution (zero mean for all DOF; standard deviation (SD) of (0.6, 2.8, 2.8) mm and (3.6, 0.9, 1.6) degrees for translations and rotations respectively; range of 3 SD based on variations observed clinically in daily setup of prostate patients.^11^, ^12^ This simulated typical interfraction variation in prostate position.

### E. Analysis

The systematic error was defined by the mean residual error for the 100 different registrations for each image set. It was used to estimate the setup error in phantom positioning, accounting for initial positioning relative to the reference CT image. The random error was taken as the difference of the systematic error from the residual errors. All arithmetic operations were performed using dual quaternions to ensure correct handling of (noncommutative) rigid body rotations and allowing simultaneous handling of both translation and rotations. To simplify the analysis, a single parameter target registration error (TRE) was used to condense the six parameters of rigid body motion into one. The TRE was determined as the maximum distance of a point on the surface of a sphere with residual translation/rotations to the initial sphere position.^(2)^ A sphere of 30 mm radius was used to approximate the surface of the prostate, centered on the reference image isocenter, which was within the prostate. This was computed using dual quaternions to combine both translational and rotational components of the residual error, and the distance denoted ${\text{TRE}}_{30}$.^(2)^ As we sought to quantify the uncertainty in imaging, the change in mean error per image was used to compare the significance of residual errors. To facilitate comparison of the mean registration error for each image, the image registration error for the standard clinical preset image was used as a baseline (see Table 1). Welch's *t*‐test was used to compare mean error between the considered image and the reference clinical preset image. For example, *t*‐tests were performed between the XVI 1360 mAs series and each other XVI series with a Bonferroni correction of n=6, such that p‐value <0.008  indicated statistical significance.

## III. RESULTS

**Figure 1:**
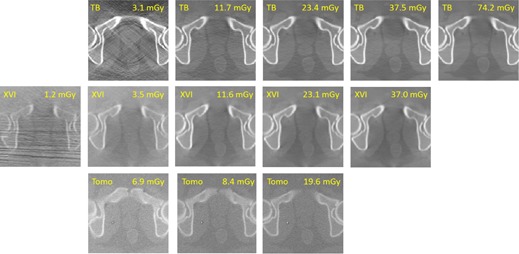
Series of images with varying dose for three IGRT imaging systems (${\text{TB}}_{\text{sharp}}$, ${\text{XVI}}_{\text{smooth}}$, and TomoTherapy). The effective dose was calculated using published in‐air dose/mAs factors.^(3)^

Slices through the prostate region of the phantom used for image registration are shown in Fig. 1 for all image sets investigated.

### A. Residual error of automatic registrations

**Figure 2:**
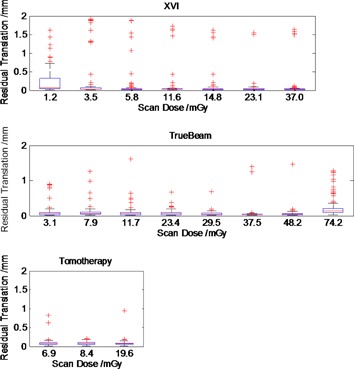
Residual translation error magnitude for ${\text{XVI}}_{\text{smooth}}$, ${\text{TB}}_{\text{sharp}}$, and TomoTherapy. Boxplots in order of increasing dose from left to right within each plot. Each boxplot represents 100 image registrations. Residual translation errors were <0.5 mm, within 1 SD.

All systems showed good registration performance, with mean residual translations less than 0.5 mm (1 σ) (Fig. 2). Over the clinical dose range, automatic registration provided similar residual errors for all imaging systems. Residual translations were <0.5 mm (1σ) for all systems. No statistical dose dependence was observed except at the lowest XVI dose and highest TrueBeam dose (Table 2).

Residual rotation errors (1σ) were 1.2°, 0.8°, and 1.9° for TrueBeam, XVI, and Tomotherapy, respectively. No strong relationship between residual rotation error and dose was observed. This can be accounted for by the approximately spherical shape of the prostate target within the matching mask, which does not have a strong rotational preference. The large spread of residual rotation error impacts on the overall quality of the automatic registration (a match with zero translational error but several degrees of rotational error is a poor match outside of the near‐spherical target). The use of the quaternion‐derived TRE metric incorporates translation and rotation in an effort to quantify the match for the six DoF.

**Figure 3:**
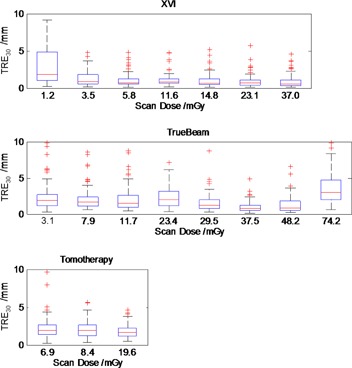
Residual ${\text{TRE}}_{30}$ for ${\text{XVI}}_{\text{smooth}}$, ${\text{TB}}_{\text{sharp}}$, and TomoTherapy. Boxplots in order of increasing dose from left to right within each plot. Each boxplot represents 100 image registrations. Failures of the automatic registration were considered when ${\text{TRE}}_{30}$ was larger than 3.6 mm. This occurred in 5%–10% of matches over the central clinical dose range. Large errors were observed for low dose on XVI and high dose on TB.

The ${\text{TRE}}_{30}$ for images acquired with imaging dose in a typical clinical range was similar between all imaging systems (Fig. 3). There was little difference in the range of ${\text{TRE}}_{30}$ results across all image sets except for XVI scans with dose less than 4 mGy and TrueBeam scans with very high dose, where ${\text{TRE}}_{30}$ increased. A pass/fail tolerance for any given automatic registration of ${\text{TRE}}_{30}>3.6\;\text{mm}$ was chosen for comparison with previous studies.^2^, ^11^ Using this tolerance the algorithm was successful in 90%–95% of registrations except for the lowest dose XVI scan (69%) (see Table 2).

**Table 2:** Consolidated results of all automatic registrations.

*Treatment System*	*Exposure (mAs)*	*Dose (mGy)*	*Reconstruction*	*Residual Translation Magnitude (mm)*	*Residual* ${\text{TRE}}_{30}$ (mm)	*% Pass Rate*	*p‐values* ^a^
	68	1.2	Standard	0.2±0.3	3.6±3.9	68	0.002^b^
	204	3.5	Standard	0.2±0.5	3.7±7.3	85	0.025
Elekta Synergy	340	5.8	Standard	0.1±0.3	2.1±4.6	91	0.719
XVI	660	11.6	Standard	0.1±0.3	2.3±5.4	91	0.682
	870	14.8	Standard	0.1±0.3	1.9±4.6	93	0.999
	1360	23.1	Standard	0.1±0.3	1.9±4.7	94	Reference
	2176	37.0	Standard	0.1±0.3	2.1±5.4	92	0.875
	89	3.1	Standard	0.1±0.1	2.5±2.2	81	0.057
	227	7.9	Standard	0.1±0.1	2.3±2.2	86	0.043
Varian	334	11.7	Standard	0.2±0.2	3.3±8.7	86	0.067
TrueBeam	669	23.4	Standard	0.1±0.1	2.9±3.7	82	0.261
	842	29.5	Standard	0.1±0.1	1.5±1.2	96	0.741
	1070	37.5	Standard	0.1±0.1	1.3±2.3	97	Reference
	1338	48.2	Standard	0.1±0.1	1.5±1.9	96	0.877
	2119	74.2	Standard	0.2±0.2	4.1±3.3	57	<0.001^b^
TomoTherapy Hi‐Art	Coarse	6.9	Standard	0.1±0.1	2.2±1.3	90	0.928
	Medium	8.4	Standard	0.1±0.0	2.1±1	93	Reference
	Fine	19.6	Standard	0.1±0.1	1.9±1.3	95	0.893

^a^ P‐values for Welch's *t*‐test of mean residual translation for the given image and the clinical preset image, with Bonferroni correction.

^b^ Statistically significant p‐values.

### B. Effect of dose and reconstruction on image quality

The image quality for the XVI, TrueBeam, and TomoTherapy images deteriorated with decreasing dose, showing increased noise. For the lowest‐dose XVI and TrueBeam images, artifacts were observed which were likely due to photon starvation (Fig. 1). However, for images in the typical range of clinical doses (10–30 mGy), similar image quality was observed based on the ability to resolve anatomical landmarks in soft tissue such as the rectal wall. 5 mm diameter low‐contrast TLD plugs in the phantom were only visible in scans with more than 23 mGy, with all systems.

**Figure 4:**
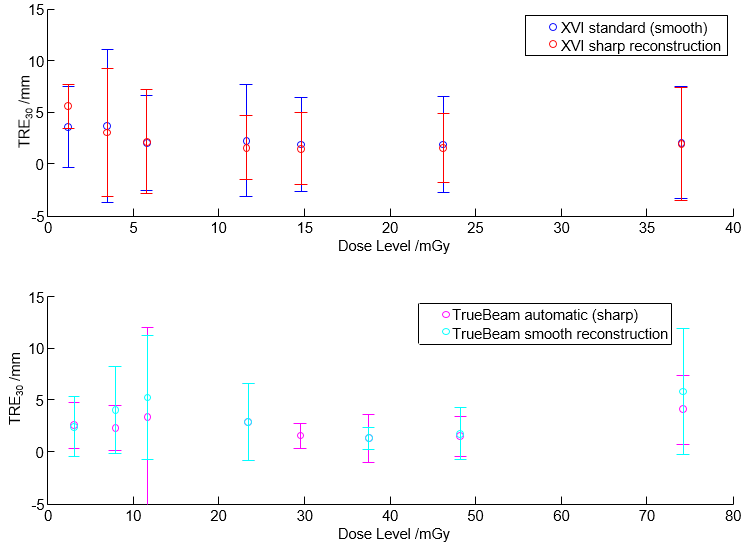
Mean residual ${\text{TRE}}_{30}$ error for smooth and sharp image reconstructions (XVI top and TrueBeam bottom). No significant difference in automatic registration was observed between the reconstructions. Error bars represent 1σ.

Reconstruction of XVI images with sharp image quality did not significantly change the automatic mean image registration results (p>0.5 for ${\text{TRE}}_{30}$, residual translations and rotations), except for the lowest dose level where sharp 1.2 mGy images exhibited worse mean automatic registration than the standard images (mean ${\text{TRE}}_{30}$ increased from 3.6 to 5.5; p<0.001). Figure 4 shows mean and standard deviation ${\text{TRE}}_{30}$ for standard and sharp reconstructions across the dose range, with no statistically significant difference.

### C. Relationship of initial and residual offsets

**Figure 5:**
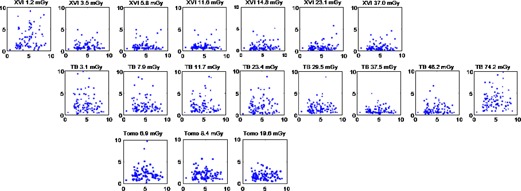
Initial misregistration (x‐axis) plotted against ${\text{TRE}}_{30}$ residual registration error (y‐axis). All axes in millimetres and represent distances on a 30 mm sphere surface. Each plot increases in dose from left to right, with approximately equal doses for positions top to bottom. The results for ${\text{XVI}}_{\text{smooth}}$ and TBsharp reconstructions are shown, however no strong trend was observed with dose for any reconstructions.

Intuitively a large initial offset would be more likely to result in a larger residual error than a small initial offset. However, no strong trend was observed regardless of dose level (Fig. 5). Registration optimization sometimes produced a result where residual errors were larger than the initial offset, particularly for small initial offsets.

## IV. DISCUSSION

Over the clinical dose range for prostate IGRT images (10–25 mGy), all three imaging systems performed similarly and within typically acceptable clinical specifications. From this phantom study, automatic image matching uncertainty is acceptable in the majority of cases (>90%) for regular targets such as the prostate, in contrast to anecdotal use of these systems.^(13)^ It is difficult to generalize this to (nonrigid) clinical patients. Additionally, no difference in automatic registration performance was found between pitch settings of TomoTherapy MVCT although there is substantial time required to obtain higher‐pitch images. The increased z‐resolution may be unnecessary for image matching purposes.

At very‐low dose (1.2 mGy) there was limited signal in the image, making manual matching quite difficult. The automatic image registration had poor results compared to higher doses; however, it still provided acceptable registrations in 69% of runs. This was despite very low visual information content for manual matching at this dose level.

The very‐high dose (>40 mGy) TB images were easily registered manually; however, the automatic registration failed in >40% of runs. The images were investigated for image quality degradation which could have affected the performance of the correlation ratio algorithm, but no definite reason was found. The following have been excluded as causes: detector panel saturation, ghosting artifacts, and aliasing artifacts from the antiscatter grid.

Škerl et al.^(14)^ investigated the role of similarity metrics on CBCT registration and found an asymmetric gradient‐based mutual information metric performed the best for CT‐CBCT registration. However, a correlation ratio metric performed similarly, with an equal rate of successful registration and they note that the correlation ratio performance improved with increased image quality (the CBCT used were reconstructed from projections more sparsely sampled than used in this study and used typically in radiotherapy). Kim et al.^(15)^ investigated similarity metrics on clinical prostate plans, and had operators review the best matches. The gradient cross correlation metric was ranked highly, with results comparable to those presented here (mean registration error 1.7±0.7 mm,96% of registrations less than 3 mm error). On a single imaging system, Cui et al.^(8)^ reported deviations of several millimeters between automatic registration algorithms on the same patient data. As smaller uncertainty was observed in the present study, it indicates that uncertainty in image registration is dominated by algorithm or similarity metric rather than imaging system.

The role of rotation corrections in radiotherapy image guidance is complicated^(16)^ and the value of rotation residuals alone may be of little meaning. Using dual quaternion distances for residual error instead of residual translation alone provides a precise way to incorporate rotations. The dual quaternion residuals have a greater scale than translations, which appear to exaggerate the registration error when considered in radiotherapy applications where rotation does not preclude dose coverage, at least for regular shaped target volumes such as prostate and lung.

Image quality comparisons between the systems were subjective due to the differing sharpness and noise present in each system's images. The MVCT/CT image registration of TomoTherapy was similar in performance to kV‐CBCT/CT image registration of conventional linac CBCT systems, using the “grey‐value” correlation ratio algorithm for automatic image registration. This was in spite of different voxel intensity distributions in the images. The algorithm is believed to use relative difference rather than absolute difference, and the contrast in each system was sufficient for the registration algorithm.

While these results are favorable to the use of automatic image registration in the clinic, phantom results alone for image uncertainty do not incorporate the many other uncertainties and errors in the treatment chain: MV‐kV beam coincidence, anatomical deformation, mobile anatomy, motion blur effects, and contrast agents. These uncertainties will also have impact on the final accuracy of the automatic image registration in the clinical workflow. The results presented in this study provide a “best‐case” baseline scenario for consideration of the imaging dose and the algorithm alone, providing guidance on these factors assuming other influencing factors are held constant.

This work has been on a prostate phantom. In situations where the contrast of the target mask might be improved, such as a lung phantom incorporating low density lung with higher density tumor, translation uncertainty would be expected to be reduced by sharpening the minima for the similarity metric.^(17)^ The fine anatomical detail present in patient images compared to bulk homogenous regions of a phantom may also improve the performance of the similarity metric and reduce uncertainty. But in practice this benefit is likely to be lost due to CBCT image quality and the nonrigid nature of patients over the treatment course, with strong contrast objects such as gold fiducial markers and calcifications often driving registrations.

The use of prefiltering projection images prior to CBCT reconstruction (for example, the XVI images use a 5 voxel median filter, ${\text{XVI}}_{\text{sharp}}$ uses no filter) had no significant impact on the automatic image registration result. Information in the image dominates over the change in sharpness/contrast provided by this filtering.

## V. CONCLUSIONS

Automatic image registration performance for three common IGRT systems was compared. The uncertainty in all the systems tested was found to be acceptable for clinical use, within the normal range of acquisition settings. This was despite differences in the image formation (beam energy, scatter, and tomographic reconstruction), and indicates images have sufficient feature for registration purposes. Outside normal settings, large uncertainties were observed at very low and very high dose levels. Results are for phantom studies, using the correlation ratio similarity metric, and therefore represent a best‐case scenario; patient images may incur higher uncertainties and review by a trained operator is necessary.

## ACKNOWLEDGMENTS

The authors would like to thank Adrian Bailey, for the loan of the phantom used in this study; and Shrikant Deshpande, Emily Flower, and Jacek Chojnowski for help with image acquisition.

## COPYRIGHT

This work is licensed under a Creative Commons Attribution 3.0 Unported License.
